# The impact of COVID-19 restrictions on HIV prevention and treatment services for key populations in South Africa: an interrupted time series analysis

**DOI:** 10.1186/s12889-024-19679-0

**Published:** 2024-09-02

**Authors:** Danwei Yao, Naomi Hill, Ben Brown, Dorian Gule, Matshidiso Chabane, Mfezi Mcingana, Kalai Willis, Vusi Shiba, Oluwasolape Olawore, Dawie Nel, Jacqueline Pienaar, Johanna Theunissen, Katherine Rucinski, Katie Reichert, Lauren Parmley, J. Joseph Lawrence, Stefan Baral, Amrita Rao

**Affiliations:** 1 Department of Epidemiology, Johns Hopkins School of Public Health, Baltimore, USA; 2 Wits RHI, University of the Witwatersrand, Johannesburg, South Africa; 3 Anova Health Institute, Cape Town, South Africa; 4 OUT LGBT Well-Being, Johannesburg, South Africa; 5 Aurum Institute, Johannesburg, South Africa; 6 TB HIV Care, Cape Town, South Africa; 7 USAID/Southern Africa, Bilateral Health Office, Pretoria, South Africa; 8 Panagora Group, Silver Spring, USA; 9 Department of International Health, Johns Hopkins School of Public Health, Baltimore, USA

**Keywords:** South Africa, Female sex workers, Gay and other men who have sex with men, Transgender women, Key populations, COVID-19, HIV, Service delivery, Interrupted time series

## Abstract

**Background:**

Key populations (KP), including men who have sex with men (MSM), female sex workers (FSW), and transgender women (TGW), experience a disproportionate burden of HIV, even in generalized epidemics like South Africa. Given this disproportionate burden and unique barriers to accessing health services, sustained provision of care is particularly relevant. It is unclear how the COVID-19 pandemic and its associated restrictions may have impacted this delivery. In this study, we aimed to describe patterns of engagement in HIV prevention and treatment services among KP in South Africa and assess the impact of different COVID-19 restriction levels on service delivery.

**Methods:**

We leveraged programmatic data collected by the US President’s Emergency Plan for AIDS Relief (PEPFAR)-supported KP partners in South Africa. We divided data into three discrete time periods based on national COVID-19 restriction periods: (i) Pre-restriction period, (ii) High-level restriction period, and (iii) After-high level restriction period. Primary outcomes included monthly total HIV tests, new HIV cases identified, new initiations of pre-exposure prophylaxis (PrEP), and new enrollments in antiretroviral therapy (ART). We conducted interrupted time series segmented regression analyses to estimate the impact of COVID-19 restrictions on HIV prevention and treatment service utilization.

**Results:**

Between January 2018 and June 2022, there were a total of 231,086 HIV tests, 27,051 HIV positive cases, 27,656 pre-exposure prophylaxis (PrEP) initiations, and 15,949 antiretroviral therapy initiations among MSM, FSW and TGW in PEPFAR-supported KP programs in South Africa. We recorded 90,457 total HIV tests during the ‘pre-restriction’ period, with 13,593 confirmed new HIV diagnoses; 26,134 total HIV tests with 2,771 new diagnoses during the ‘high-level restriction’ period; and 114,495 HIV tests with 10,687 new diagnoses during the after high-level restriction period. Our Poisson regression model estimates indicate an immediate and significant decrease in service engagement at the onset of COVID-19 restrictions, including declines in HIV testing, treatment, and PrEP use, which persisted. As programs adjusted to the new restrictions, there was a gradual rebound in service engagement, particularly among MSM and FSW. Towards the end of the high-level restriction period, with some aspects of daily life returning to normal but others still restricted, there was more variability. Some indicators continued to improve, while others stagnated or decreased.

**Conclusion:**

Service provision rebounded from the initial shock created by pandemic-related restrictions, and HIV services were largely maintained for KP in South Africa. These results suggest that HIV service delivery among programs designed for KP was able to be flexible and resilient to the evolving restrictions. The results of this study can inform plans for future pandemics and large-scale disruptions to the delivery of HIV services.

**Supplementary Information:**

The online version contains supplementary material available at 10.1186/s12889-024-19679-0.

## Introduction

Coronavirus disease 2019 (COVID-19) has been one of the most significant public health challenges of the modern era, and as of 2023, the number of those infected with SARS-CoV-2 continues to rise globally [1]. COVID-19 has resulted in significant morbidity and mortality and, in many instances, disrupted the normal functioning of critical health systems [2]. To stem the spread of infection, many countries implemented policies and mitigation strategies that have altered the functioning of daily life, including but not limited to travel restrictions, school and business closures, and physical distancing [3]. The depth and breadth of these approaches have varied across countries, with varying degrees of success in reducing COVID-19 transmission at a population level [4].

In South Africa, a full national lockdown was announced in late March 2020, 23 days after the first confirmed case of COVID-19 was detected in the country [5]. This full national lockdown meant that gatherings were prohibited, and restaurants and schools were closed. “Non-essential” individuals were only allowed to leave their homes to access health and social services and pick up essential goods. While healthcare workers and pharmacy and laboratory personnel were exempt from these restrictions to provide services, most of daily life changed as a result and altered the context that health services needed to function within [6, 7]. As an example, healthcare workers were reliant on employer issue of specific healthcare worker permits to be exempt from restrictions. Despite specific permits, police and other official authorities still restricted and limited movement of healthcare workers in certain instances, limiting capacity to provide services. The South African government defined five different COVID-19 alert levels to determine restrictions. Over the following two years, implementation of restrictions continued to vary with changing transmission levels [6, 7].

Amidst the public health challenges created by COVID-19, South Africa also faces an ongoing HIV crisis. In South Africa, close to 1 in 5 adults are living with HIV, and more than 231,000 individuals are newly infected each year [8], necessitating the delivery of treatment to more than 7.5 million people living with HIV and prevention services to millions more [9]. HIV remains the leading cause of death in the country despite biomedical advances that have led to earlier diagnoses and lifelong effective clinical management through treatment [10].

Even with the incredibly high HIV risk among the general population in South Africa, key populations including men who have sex with men (MSM), female sex workers (FSW), and transgender women experience a disproportionate burden of HIV. HIV incidence is up to 25 times higher among MSM and up to 26 times higher among FSW compared to other adults of reproductive age [11]. Key populations face myriad individual, social, and structural factors that put them at increased risk of HIV acquisition [12]. The same stigma, discrimination, and criminalization that puts these groups at heightened risk can challenge access to HIV services [12].

Barriers to accessing HIV services in public health clinics among key populations have historically included a range of factors, including but not limited to misalignment of clinic hours or location of services with need, risk or fear of arrest due to criminalization of sex work, fear of disclosure of HIV or population membership, and violence from partners, community members, and others [13]. HIV service delivery in South Africa for key populations through the United States President’s Emergency Plan for AIDS Relief (PEPFAR)-funded programs has long attempted to address these barriers by including tailored delivery strategies to optimize quality, satisfaction, and efficiency of care. During the COVID-19 pandemic, implementing partners carefully monitored performance data to track service delivery interruptions and respond accordingly. Quantitative, analytic research approaches can help to substantiate what was being observed by those providing services and assess to what extent COVID-19 associated restrictions may have directly impacted delivery, specifically to populations with the greatest need [14].

The impact of COVID-19 on HIV services has been examined in multiple settings, including in South Africa, though less is known about the specific impact of the pandemic on HIV prevention and treatment service engagement among key populations [15]. While we now, in many settings, have access to effective vaccines to prevent infection with SARS-CoV-2 and mitigate the severity of the disease, there is a need to understand how well HIV services were sustained as part of PEPFAR-funded services for key populations to inform future emerging infectious disease or other public health crises. In this analysis, we aim to describe patterns of engagement in HIV prevention and treatment services among key populations in South Africa and assess the impact of different COVID-19 restriction levels on this engagement using an interrupted time series approach.

## Methods

### Study design: implementation of key population programs in South Africa

In this analysis, we utilized routine program data collected through the PEPFAR bilateral initiative in South Africa. In brief, this initiative includes a partnership with the Government of South Africa and the National and Provincial Departments of Health, which work directly to coordinate HIV service provision with implementing partners funded through both the Centers for Disease Control and Prevention (CDC) and the United States Agency for International Development (USAID) [16–18]. In this analysis, data were provided by both CDC- and USAID-funded implementing partners.

In September 2021, the Key Populations Investment Fund (KPIF), a globally launched investment program aiming to increase access to HIV services for key populations ended in South Africa [19]. KPIF funding was used to both directly provide testing, prevention, and treatment services and was also used to support community mobilization. The inflection point where KPIF ended, which relates to the availability of funding to support provision of services, has been noted in the figures, and we visually inspected the data in the context of KPIF closure.

### Study population

Routinely collected data were available for MSM, FSW, and transgender women (TGW). MSM in these analyses included gay and other men who have sex with men receiving HIV prevention or treatment services from Aurum Institute (CDC) and Anova Health Institute and OUT/LGBT Well-Being (USAID). FSW included women 18 years and older who sell sex and received HIV services from TB HIV Care (CDC) and Wits Reproductive Health Institute (USAID). TGW included women who were assigned male sex at birth and received services from Wits Reproductive Health Institute (USAID) [17, 20, 21]. These implementing partners received funding for programs and service delivery through a subagreement with FHI 360.

### Outcomes

Outcome data were derived from programmatic indicators routinely reported by implementing partners as part of standard PEPFAR program monitoring procedures. Data were abstracted as monthly counts for each population either from program files shared directly by implementing partners or from InfoLink, a database platform where routine program data are compiled from partners for reporting purposes. Data were accessible through InfoLink because of an ongoing collaboration between the study team and FHI360 [22]. The primary outcomes included 1) monthly number of individuals who received HIV testing services and received their results (HTS_TST), 2) monthly number of new HIV cases identified, or case finding (HTS_TST_Pos), 3) monthly number of new pre-exposure prophylaxis (PrEP) initiations (PrEP_NEW), and 4) monthly number of individuals who newly started/enrolled on antiretroviral therapy (ART) (TX_NEW). Based on data availability, we accessed data across different time horizons for each population.

### COVID-19 restrictions

Stringency or severity of COVID-19 restrictions was assessed based on a review of publicly available information disseminated by the Government of South Africa. Restrictions by the Government were implemented using an alert level approach, which aligned with “the level of infections and rate of transmission, the capacity of health facilities, and the extent of the implementation of public health interventions and the economic and social impact of continued restrictions.” The alert system ranged from 1 through 5, where alert level 1 represented a low level of spread and a high level of health system capacity, and alert level 5 represented a high level of spread and a low level of health system capacity. A summary of the restrictions corresponding to the alert levels are reported below [23]:

- Level 5: Only essential services, restricted times for public transportation, stay-home order and no inter-provincial movement of people.
- Level 4: Some industries resume activity (agriculture, waste management, information technology services), public transportation allowed with restricted capacity, curfew from 8 PM – 5 AM and limited inter-provincial movement.
- Level 3: Additional industries resume activity (take-away restaurants, automotive manufacturing, government services, most retail, etc.), public transportation allowed with restricted capacity, no curfew, but ongoing limited inter-provincial movement.
- Level 2: All retail, construction, domestic work, manufacturing and government services resume activity, domestic air travel and car rental allowed, movement permitted between provinces at level 1 or level 2.
- Level 1: All sectors resume activity, all modes of transportation resume with strict hygiene practices enforced, movement between provinces allowed with some restrictions on international travel.

For these analyses, we grouped data into three discrete periods based on the level of restrictions that were implemented. Data provided prior to March 2020, or pre-pandemic, comprised the “pre-restriction” period. The second period was classified as the “high-level restriction period” when alert levels three to five were in place (March 2020-August 2020), and corresponded to low to moderate health system readiness. Finally, the third period, including alert level two, alert level one, and lifted restrictions (September 2020-June 2022), was classified as the “after high-level restriction period,” corresponding to high health system readiness.

### Statistical analysis

The number of individuals served by each program were reported for each key population, and summary statistics on the primary outcomes were described by intervention period (high-level restriction period and after high-level restriction period). We conducted interrupted time series segmented regression analyses to assess the impact of COVID-19 restrictions on our primary HIV prevention and treatment outcomes. We fit Poisson or negative-binominal regression models based on dispersion of the data for each population separately. An assumption of the Poisson distribution is that the mean is equal to the variance. In cases where the variance was larger than the mean, we utilized the negative binomial model and incorporated an additional term to account for the excess variance [24]. We fit one model per population per outcome (3 populations × 4 outcomes = 12 total models). Models included a variable to account for time since the start of the study period, two dummy variables indicating high-level and after-high-level restriction periods and their interaction terms with their restriction implementation time period, respectively. This approach takes account of different levels of restrictions and allows evaluation of the immediate effect of restrictions at different time points by centering time at that time point. The statistical model used in this paper took the following form:

$$Log\left({Y}_{t}\right)={\upbeta }_{0}+{\upbeta }_{1}Time+{\upbeta }_{2}High+{\upbeta }_{3}AfterHigh+{\upbeta }_{4}High*{t}_{h}+{\upbeta }_{5}AfterHigh*{t}_{l}+ \varepsilon$$

where Y represents the count of the specified outcome for each calendar month,${e}^{{\upbeta }_{0}}$ represents the level or the count of the outcome at T = 0 (start of the study period). ${e}^{{\upbeta }_{1}}$ represents the change in the outcome associated with time during the pre-restriction period (pre-restriction trend), Time denotes the number of months since the start of study period. ${e}^{{\upbeta }_{2}}$ and ${e}^{{\upbeta }_{3}}$ represent the immediate level changes following implementation of the high-level restriction period and the after high-level restriction period relative to the pre-restriction period, respectively. ${t}_{h}$ is the number of months since high level restrictions implemented, ${t}_{l}$ represents the number of months since the start of after high-level restriction period, ${e}^{{\upbeta }_{4}}$ and ${e}^{{\upbeta }_{5}}$ represent the averaged trend change following implementation of the high-level restriction and the after high-level restriction period relative to the pre-restriction period, respectively.

From the model, we estimated the immediate impact of COVID-19 restriction implementation by looking at the coefficient of the restriction period variable (${e}^{{\upbeta }_{2}}$ for high and ${e}^{{\upbeta }_{3}}$ for after high). We evaluated the post-restriction period trend for each outcome by adding together the coefficients associated with time and time-restriction interaction (${e}^{{\upbeta }_{1}+{\upbeta }_{4}}$ for high, ${e}^{{\upbeta }_{1}+{\upbeta }_{5}}$ for after high).

Newey-West standard errors with autocorrelation up to 6 lags were used within our models to account for serial autocorrelations and heteroskedasticity [25–27]. We performed all analyses in R 4.0.4 (R Foundation for Statistical Computing, Vienna, Austria; appendix).

To adjust for seasonal changes in clinic activity that have a repeating pattern with fixed frequency (e.g., holiday periods), we used a combination of sine and cosine functions to model the seasonal pattern [28]. In our case, the frequency of sine and cosine functions corresponded to the length of the seasonal cycle (12 months). The coefficient of these terms determines the strength of the seasonal pattern (amplitude), while the phase of the functions determines the timing of the seasonal pattern within the cycle. Acknowledging the inherent variability in the pre-intervention period model fitting, we introduced an additional step to enhance the interpretability and realism of the level that could conceivably have been reached for some of these indicators. Recognizing that certain indicators might exhibit unrealistic trends due to this variability, we incorporated an asymptotic line. This line serves as a cap, setting the maximum realistic value for these indicators.

### Programmatic adaptations to service delivery

To supplement the quantitative results of these analyses, programmatic details around adaptations to service delivery made during the COVID-19 pandemic were documented via a brief online questionnaire administered initially in October 2020. The questionnaire included questions regarding populations served, the type of HIV services provided, implementation strategies to ensure continuity of service provision. Specific adaptations such as new delivery methods and new strategies in response to COVID-19 were also recorded. These data were updated in November 2021; a spreadsheet was shared with each implementing partner to assess any further adaptations or innovations following the initial 2020 survey.

### Ethics

All data included in these analyses were collected for the purposes of routine program monitoring and were reported in aggregate. Analyses did not include personal identifiable information and were thus classified as non-human subjects research (JHU IRB0007442).

## Results

Data were available for MSM from June 2019 to June 2022, for FSW from January 2018 to March 2022 and for TGW from November 2018 to March 2022. A total of 231,086 HIV tests were reported among MSM, FSW, and TGW across the study period. There were 7,866 (6.8%) positive HIV tests for MSM. During this same period, there were a total of 38,981 PrEP initiations and 8,422 ART initiations. Among FSW, there were 18,049 (16.7%) positive HIV tests reported among FSW. There were 27,656 PrEP initiations and 15,949 ART initiations. Among TGW, there were 1,136 (15.9%) positive HIV tests reported from January 2019 through March 2022 among TGW. There were 2,054 PrEP initiations and 935 ART initiations.

Ninety thousand four hundred fifty-seven total HIV tests were reported during the ‘pre-restriction’ period, with 13,593 confirmed positive cases. From March 2020 to August 2020, the ‘high-level restriction’ period, 26,134 total tests, and 2,771 positive cases were reported. During the ‘after high-level restriction period,’ 114,495 HIV tests and 10,687 cases were reported.

### High-level restriction period: March 2020-August 2020

As detailed below for each indicator, the segmented regression analysis substantiated a sharp drop across the HIV indicators among all key population groups at the implementation of Level 5 national COVID-19 restrictions in March 2020.

#### Men who have sex with men

**Fig. 1:**
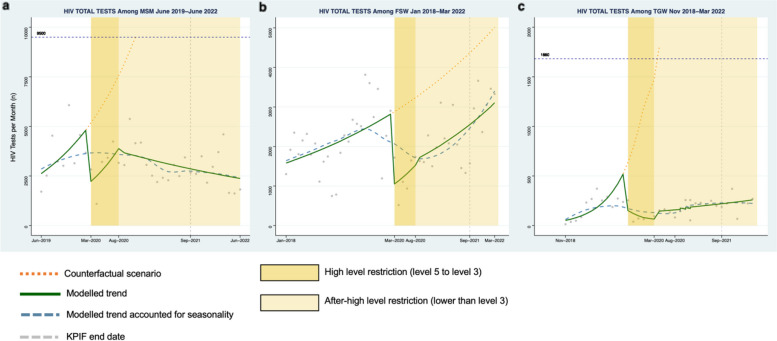
Monthly counts, trends and fitted segmented regression models of HIV total tests for Men who have sex with men (**a**), Female sex workers (**b**), and Transgender women (**c**) in South Africa

**Fig. 2:**
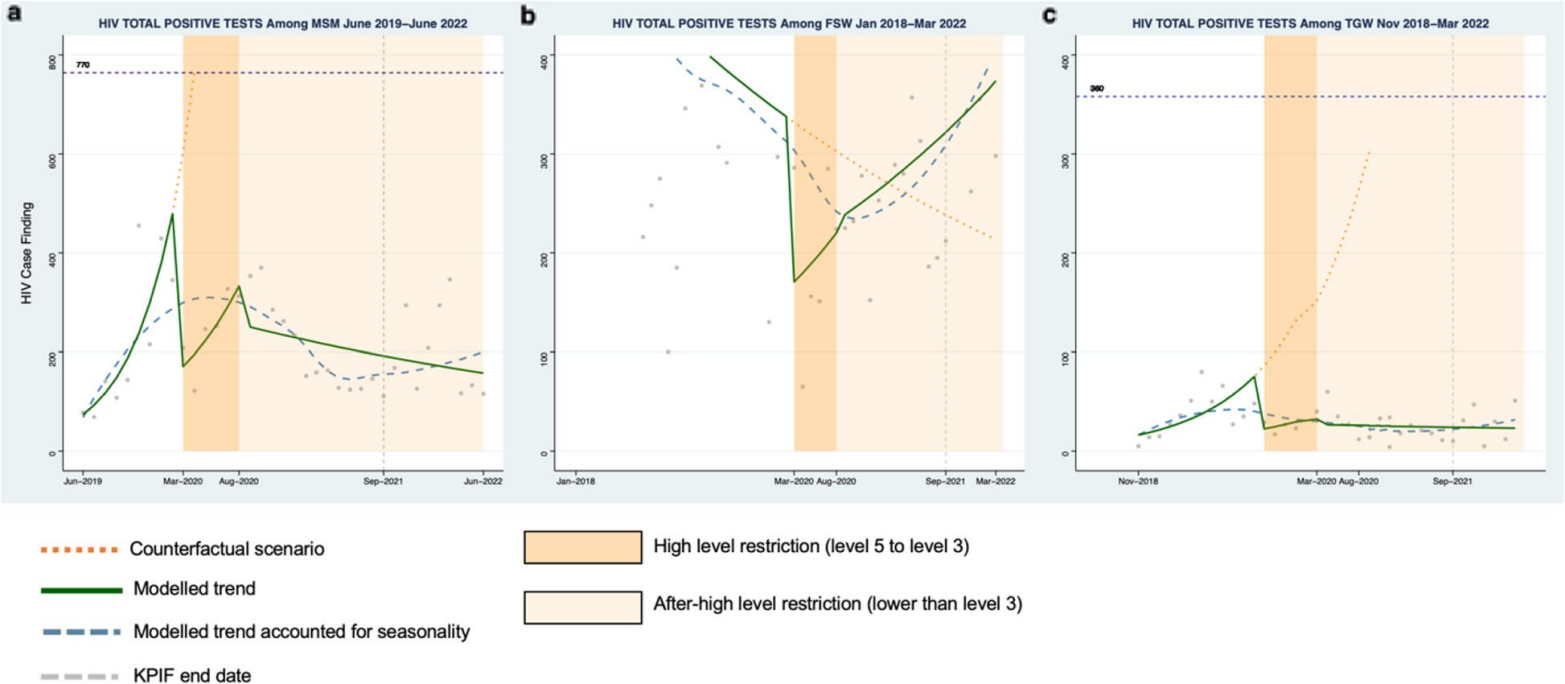
Monthly counts, trends and fitted segmented regression models of HIV positive cases for Men who have sex with men (**a**), Female sex workers (**b**), and Transgender women (**c**) in South Africa

**Fig. 3:**
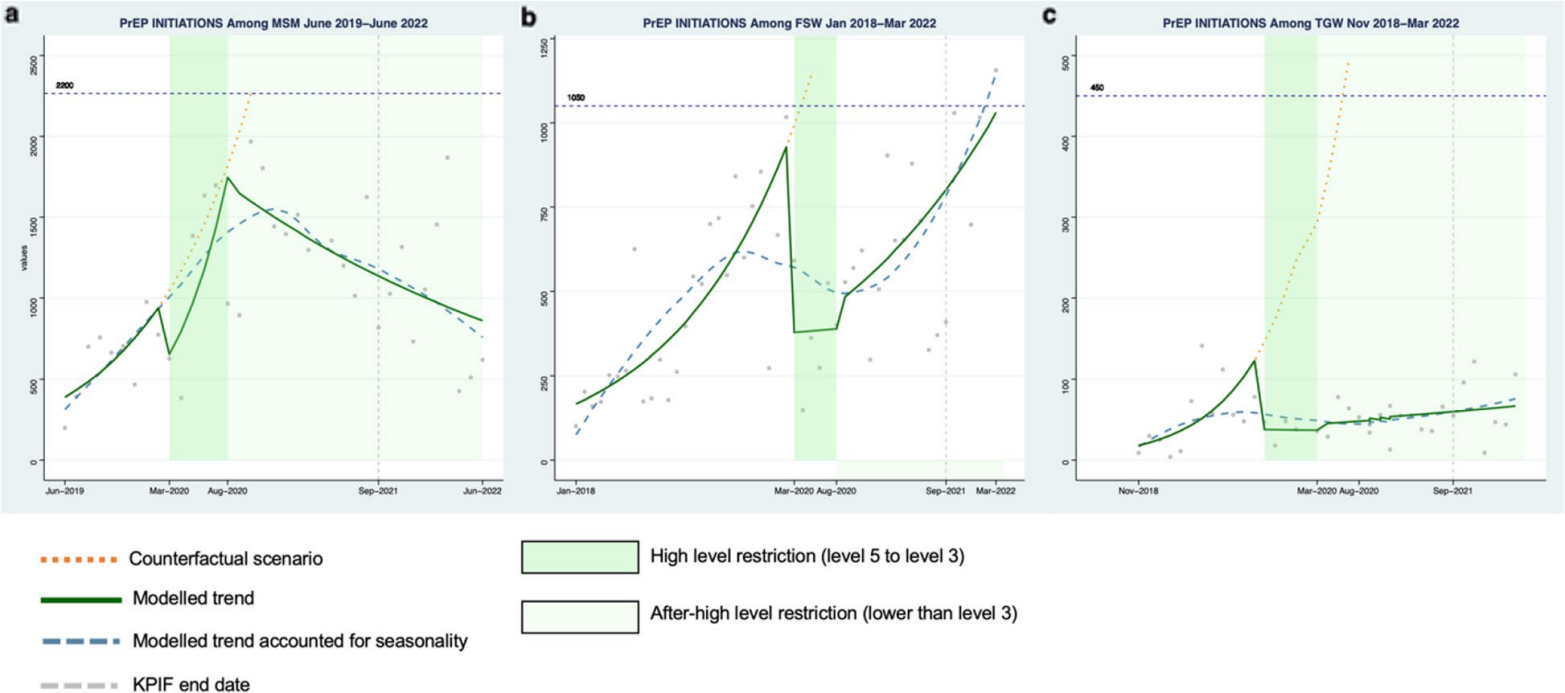
Monthly counts, trends and fitted segmented regression models of PrEP initiations for Men who have sex with men (**a**), Female sex workers (**b**), and Transgender women (**c**) in South Africa, PrEP = pre-exposure prophylaxis

**Fig. 4:**
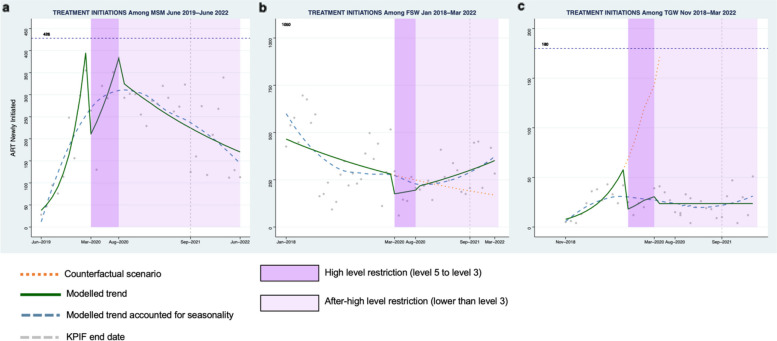
Monthly counts, trends and fitted segmented regression models of ART initiations for Men who have sex with men (**a**), Female sex workers (**b**), and Transgender women (**c**) in South Africa, ART = antiretroviral therapy

Among MSM, a 59% decrease in the number of HIV tests was observed during the first month of high-level restrictions (Fig. 1a, Risk Ratio (RR): 0.41, 95% Confidence Interval (CI): 0.27–0.64) compared to the pre-restriction period. Decreases were also seen for HIV case-finding (Fig. 2a, RR: 0.31, 95% CI: 0.21–0.46), PrEP initiation (Fig. 3a, RR: 0.57, 95% CI: 0.34–0.96), and ART initiation (Fig. 4a, RR: 0.47, 95% CI: 0.34–0.66). Starting in May 2020, all indicators demonstrated a moderate rebound following the initial drop in service engagement (Table 1). For example, HIV testing showed a gradual positive trend during this period (RR: 1.12 95% CI: 0.97–1.28).

**Table 1:** Poisson segmented regression models adjusting for seasonality of COVID-19 restrictions on PEPFAR-supported HIV services for key populations in South Africa

	**Level change associated with high level** ${{\varvec{e}}}^{{{\varvec{\upbeta}}}_{2}}$ **RR (95% CI)**^a^	**Level change associated with after high level** ${{\varvec{e}}}^{{{\varvec{\upbeta}}}_{3}}$ **RR (95% CI)**^a^	**Pre-restriction trend** ${{\varvec{e}}}^{{{\varvec{\upbeta}}}_{1}}$ **RR (95% CI)**^a^	**Trend change associated with high level** ${{\varvec{e}}}^{{{\varvec{\upbeta}}}_{1}+{{\varvec{\upbeta}}}_{4}}$ **RR (95% CI)**^a^	**Trend change associated with after high level** ${{\varvec{e}}}^{{{\varvec{\upbeta}}}_{1}+{{\varvec{\upbeta}}}_{5}}$ **RR (95% CI)**^a^
**HIV testing**					
MSM	0.41 ( 0.27, 0.64)	0.49 ( 0.27, 0.90)	1.08 ( 1.02, 1.14)	1.12 ( 0.97, 1.28)	0.98 ( 0.96, 1.00)
FSW	0.35 ( 0.17, 0.69)	0.52 ( 0.35, 0.76)	1.02 ( 1.01, 1.04)	1.08 ( 1.06, 1.09)	1.03 ( 1.03, 1.04)
TGW	0.36 ( 0.09, 1.41)	0.1 ( 0.03, 0.33)	1.23 ( 1.1, 1.38)	0.81 ( 0.6, 1.09)	1.03 ( 1.00, 1.06)
**Positive tests**					
MSM	0.31 ( 0.21, 0.46)	0.13 ( 0.07, 0.25)	1.26 ( 1.21, 1.32)	1.14 ( 0.97, 1.34)	0.98 ( 0.96, 1.00)
FSW	0.48 ( 0.24, 0.96)	0.77 ( 0.51, 1.16)	0.98 ( 0.96, 1.00)	1.05 ( 1.02, 1.09)	1.03 ( 1.02, 1.03)
TGW	0.76 ( 0.50, 1.16)	4.63 ( 0.14,158.3)	1.42 ( 0.83, 2.42)	1.74 ( 1.23, 2.47)	0.80 ( 0.58, 1.09)
**PrEP**					
MSM	0.57 ( 0.34, 0.96)	0.93 ( 0.49, 1.77)	1.12 ( 1.04, 1.20)	1.22 ( 1.04, 1.43)	0.97 ( 0.95, 0.99)
FSW	0.41 ( 0.21, 0.78)	0.33 ( 0.23, 0.48)	1.07 ( 1.05, 1.09)	1.01 ( 0.85, 1.19)	1.04 ( 1.01, 1.07)
TGW	0.31 ( 0.14, 0.68)	0.15 ( 0.05, 0.44)	1.19 ( 1.10, 1.29)	0.99 ( 0.70, 1.40)	1.02 ( 0.98, 1.06)
**ART**					
MSM	0.47 ( 0.34, 0.66)	0.15 ( 0.10, 0.21)	1.34 ( 1.3, 1.39)	1.13 ( 0.99, 1.28)	0.97 ( 0.95, 0.99)
FSW	0.61 ( 0.31, 1.21)	0.85 ( 0.53, 1.38)	0.98 ( 0.96, 1.0)	1.02 ( 0.84, 1.25)	1.03 ( 0.99, 1.06)
TGW	0.28 ( 0.12, 0.63)	0.17 ( 0.06, 0.44)	1.20 ( 1.1, 1.31)	1.14 ( 0.82, 1.57)	1.00 ( 0.97, 1.04)

^a^Autocorrelation addressed using Newey-West standard errors to estimate the confidence interval with lag up to 6

#### Female sex workers

Among FSW, there was a 65% decrease in HIV testing at the start of high-level restrictions (Fig. 1b, RR: 0.35, 95% CI: 0.17–0.69). Similarly, there were declines observed for HIV case finding (Fig. 2b, RR:0.48, 95% CI: 0.24–0.96), PrEP initiation (Fig. 3b, RR: 0.41, 95% CI: 0.21–0.78), and ART initiation (Fig. 4b, RR: 0.61, 95% CI: 0.31–1.21). There was a similar rebound in outcomes as was seen among MSM during this period, with HIV testing gradually improving over time for FSW (RR: 1.08, 95% CI: 1.06–1.09).

#### Transgender populations

Findings were similar among TGW with a 64% (Fig. 1c, RR: 0.36, 95% CI: 0.09–1.41) decrease in HIV testing, a 23% (Fig. 2c, RR: 0.76: 95% CI:0.50–1.16) decrease in positive cases, a 69% (Fig. 3c, RR: 0.31, 95%CI: 0.14–0.68) decrease in PrEP initiations, and a 72% (Fig. 4c, RR: 0.28, 95% CI:0.12–0.63) decrease in ART initiations. Among TGW, there was a similar gradual increase in each of the indicators over time except for HIV testing, which saw a decline in the trend over time during this period.

Additional changes in the key outcomes over time are documented in Table 1.

### After high-level restriction period: September 2020-June 2022

After the high-level restriction period, we observed a decline in the level of all HIV indicators across the various key population groups. Patterns in trends varied across indicators and populations.

#### Men who have sex with men

For MSM, a 51% decline in the number of HIV tests was detected at the start of the after-high level restriction period relative to the level during the pre-restriction period (April 2020) (Fig. 1a, RR: 0.49, 95%CI 0.27–0.90). Similar reductions were also observed among other indicators (Table 1). Thereafter, the segmented analysis revealed a decrease of 2% per month (Fig. 1a, RR: 0.98, 95%CI: 0.96–1.00) in HIV tests. Moderate declines over time were also found in HIV case finding (Fig. 2a, RR:0.98, 95%CI: 0.96–1.00), PrEP initiation (Fig. 3a, RR: 0.97, 95% CI: 0.95–0.99), and ART initiation (Fig. 4a, RR: 0.97 95% CI: 0.95–0.99).

#### Female sex workers

Among FSW, the level of HIV testing declined at the start of the after high-level restriction period compared with the pre-restriction period (Fig. 1b, RR: 0.52, 95%CI: 0.35–0.76). The other primary indicators showed a similar pattern (Table 1). During this period, we observed HIV testing numbers gradually recovered by 3% per month (RR: 1.03, 95%CI: 1.03–1.04). Positive increases per month were also seen in other indicators: HIV case finding (Fig. 2b, RR:1.03, 95%CI: 1.02–1.03), PrEP initiations (Fig. 3b, RR: 1.04, 95%CI: 1.01,1.07), and ART initiations (Fig. 4b, RR: 1.03, 95%CI: 0.99–1.06).

#### Transgender populations

Among TGW, there was a significant decrease in the level of HIV testing at the start of the after high-restriction period (Fig. 1c, RR: 0.1, 95% CI: 0.03, 0.33). Decreases were also seen in PrEP initiations and ART initiations (Table 1). After that, HIV testing consistently improved during this period (Fig. 1c, RR: 1.03, 95%CI: 1.00–1.06), while other indicators stayed at a constant level with no significant increase (Table 1).

#### Programmatic adaptations to service delivery

During this period, implementing partners enacted several modifications to HIV community outreach programs to adapt to COVID-19 restrictions. Strategies varied across partners and population groups. Some example efforts that were made to provide more virtual support included utilizing mobile online communication (WhatsApp, Facebook groups) and a bulk SMS platform to inform clients of updates and changes to clinic services, times, and locations. Concurrently, self-testing, multi-month dispensing, and home-based ART-delivery along with telemedicine appointments were introduced and offered to clients during this time. For TGW identified by service providers to have increased housing insecurity and who were forced into shelters during the pandemic, mobile HIV services were brought to these shelters to ensure sustained services. For FSW, who were unable to work and earn an income as a result of restrictions, implementing partner teams solicited and provided food parcels. Additional innovations included small group meetings with peer navigators and outreach based on where clients live rather than at centralized locations historically dictated by hotspots (bars, brothels, clubs etc.). For MSM, ambassadors from different wards hosted community advisory board meetings to identify PrEP needs and provided additional support in transferring of clients to other clinics to continue provision of care. Other peer navigation teams were formed for further home visits, HIV testing, and PrEP deliveries. Implementing partners were able to reintegrate their standard community-based activities and mobile services, as well as create small group peer meetings, form peer navigation teams, and utilize community ambassadors from different wards to host community advisory meetings to support local client needs.

## Discussion

In this analysis, we evaluated the impact of national-level COVID-19 restrictions on HIV prevention and treatment service utilization for key populations accessing PEFPAR-supported HIV services in South Africa utilizing an interrupted time series approach. At the onset of pandemic restrictions, we observed an immediate and pronounced decrease in service engagement, including declines in the number of individuals accessing HIV testing, treatment and PrEP that persisted for MSM, FSW, and TGW. As programs began to adjust and adapt to restrictions, there was a gradual rebound in service engagement, particularly among MSM and FSW. At the end of the high-level restriction period, with most returning to normal, but some movement and daily life still restricted, there was more variation across the different indicators. Some indicators continued to make improvements and increase, others stagnated, and still others decreased. Taken together, these findings largely highlight the flexibility and resiliency of HIV service delivery and the maintenance of HIV services for those with the greatest need during the pandemic in South Africa.

Consistent with what has been described elsewhere in sub-Saharan Africa, we found an immediate impact on the uptake of critical HIV services following the implementation of COVID-19 restrictions. A multi-country study among MSM involving 20 countries revealed that 30% of 10,654 surveyed individuals experienced interruptions to in-person HIV testing, with 55% reporting interruptions to HIV self-testing [4]. A study using routine data across populations from public sector clinics in South Africa saw similar declines in access to these same services, with HIV testing being among the most affected with considerable variation by province [29]. A cohort study in the Western Cape Province of South Africa found decreases in the number of PrEP visits among women attending antenatal care [30]. A review of studies across South Africa found reported decreases in HIV testing, positive HIV tests, and initiation of ART [31]. While delivery of HIV services remained essential during even the most stringent Level 5 restrictions, the changing context of daily life placed additional burden on programs in the early months of the pandemic. Some of these changes included suspension of certain in-person services to promote physical distancing, staff shortages due to the need for quarantine for exposed individuals and isolation for those infected with COVID-19, closure of community hotspots, including bars, brothels, and nightclubs where outreach for key populations often occurs, suspension of peer outreach, and the confluence of COVID-19 and HIV related stigma [14]. Despite these closures, the need for services remained and in some instances was likely heightened. The pandemic pushed already stigmatized and hidden hotspots and areas of congregation further underground, increasing the likelihood of violence, discrimination and harassment from clients, partners, police, and others [32].

Despite the initial declines observed associated with pandemic restrictions, a gradual rebound was seen across most indicators over time, reflecting both an easing of restrictions and efforts by partners to ensure the continuity of services. A large-scale study looking at the impact of COVID-19 lockdown on HIV care across 65 South African primary care clinics similarly found that while there were immediate declines associated with pandemic restrictions, there was a measured but ongoing observed improvement in HIV testing and ART initiations as restrictions began to ease [29]. Other studies also found that HIV services were resilient to the shocks and interruptions created by COVID-19 restrictions, especially in the months immediately after the beginning of the pandemic with easing restrictions [6, 33].

We considered two key programmatic shifts that occurred during this period: program-driven adaptations to services to maintain delivery during the pandemic and the end of KPIF, a key funding mechanism that provided additional funds for key populations programs with a focus on capacity building of key population-led organizations and evaluated their relevance by visually inspecting the Figures. As documented in the Results section, implementing partners contributing data to these analyses made changes and adaptations to better support those accessing services during the pandemic. Further research is needed to understand which of these adaptations and implementation strategies proved most useful in sustaining provision of services, but it is clear that creative adaptations like the ones mentioned here will be critical for future pandemics or other disruptive events. KPIF was primarily intended to provide additional capacity for key populations organizations and test certain innovations. Qualitatively, we did not observe a considerable impact of the conclusion of KPIF on the primary HIV indicators, but this was not the primary analytic question of this study.

There were two key strengths of this study. First, we leveraged routinely collected program data for these analyses, which meant that we were able to elucidate patterns in HIV service engagement use and better understand fluctuations in that use at no additional cost or burden to the program or to service users. Second, these data are largely representative of key populations accessing PEPFAR-funded services in South Africa, as we did not do any sampling or selective inclusion. This is particularly important as representative data for key populations are often difficult to collect, given high levels of mobility and systemic marginalization and discrimination and associated fear and distrust.

There were several limitations of this study. First, a key assumption of the statistical models is that COVID-19 restrictions in practice changed in alignment with the official alert levels put forward by the Government. In practice, there may have been variability in when different restrictions went into place and how this impacted daily life. For the purposes of the interrupted time series, this was a simplifying assumption to establish a clear-cut point but one that we feel does broadly align with both perceived and actual changes that occurred. Second, we combined data across different implementing partners for a single population and assumed that patterns would be similar across the programs. There could be variability by implementing partner depending on the size of the program, for example based on the number of clients served or number of staff involved, or the geographic area. Thirdly, while our seasonal adjustment aims to account for known patterns such as reduced activity during holiday periods, the limited data duration may affect the precision of these adjustments. Finally, there was a significant amount of relocation with people moving out of urban centers during the pandemic, and the underlying population may have been changing over time. Though we were unable to assess changes to population characteristics, as we did not have access to individual-level demographic and behavioral data, we do not anticipate that the distribution of these characteristics was changing dramatically on average over time.

HIV service utilization among key populations in South Africa was severely impacted in the initial months of the pandemic by stringent restrictions intended to stem the spread of SARS-CoV-2. As restrictions eased, and as programs adapted to the context of changing daily life, there was a gradual rebound in the uptake of services across populations. These results highlight the resiliency and dynamicity of PEPFAR-funded implementing partners in generating creative solutions, including virtual support, multi-month dispensing and home delivery of HIV testing and ART, and amplified peer navigation, to maintain HIV services during the COVID-19 pandemic. As of the end of the study period, however, the level of uptake of most services had not yet returned to pre-pandemic levels. There is an ongoing need to come up with new strategies to get key populations in need of HIV services re-engaged in care and to evaluate existing strategies to understand which are most effective at ensuring continuity of care. Using these results and the results from future studies, we can begin to develop plans for future pandemics and large-scale disruptions to the delivery of HIV services.

### Supplementary Information

Supplementary Material 1

## Data Availability

The datasets used during the current study are available from the corresponding author on reasonable request.

## Abbreviations

COVID-19: Coronavirus disease 2019
MSM: Men who have sex with men
FSW: Female sex workers
TGW: Transgender women
PEPFAR: United States President’s Emergency Plan for AIDS Relief
CDC: Centers for Disease Control and Prevention
USAID: The United States Agency for International Development
KPIF: Key Populations Investment Fund
ART: Antiretroviral therapy
