# Supplementary material for: The impact of COVID-19 restrictions on HIV prevention and treatment services for key populations in South Africa: an interrupted time series analysis
Source: BMC Public Health. 2024 Sep 2;24:2386. doi: 10.1186/s12889-024-19679-0 (PMC11370299; doi:10.1186/s12889-024-19679-0)

# Supplemental Appendix

Figure S1. Provincial geographic mapping of service delivery by CDC- and USAID-implementing partners


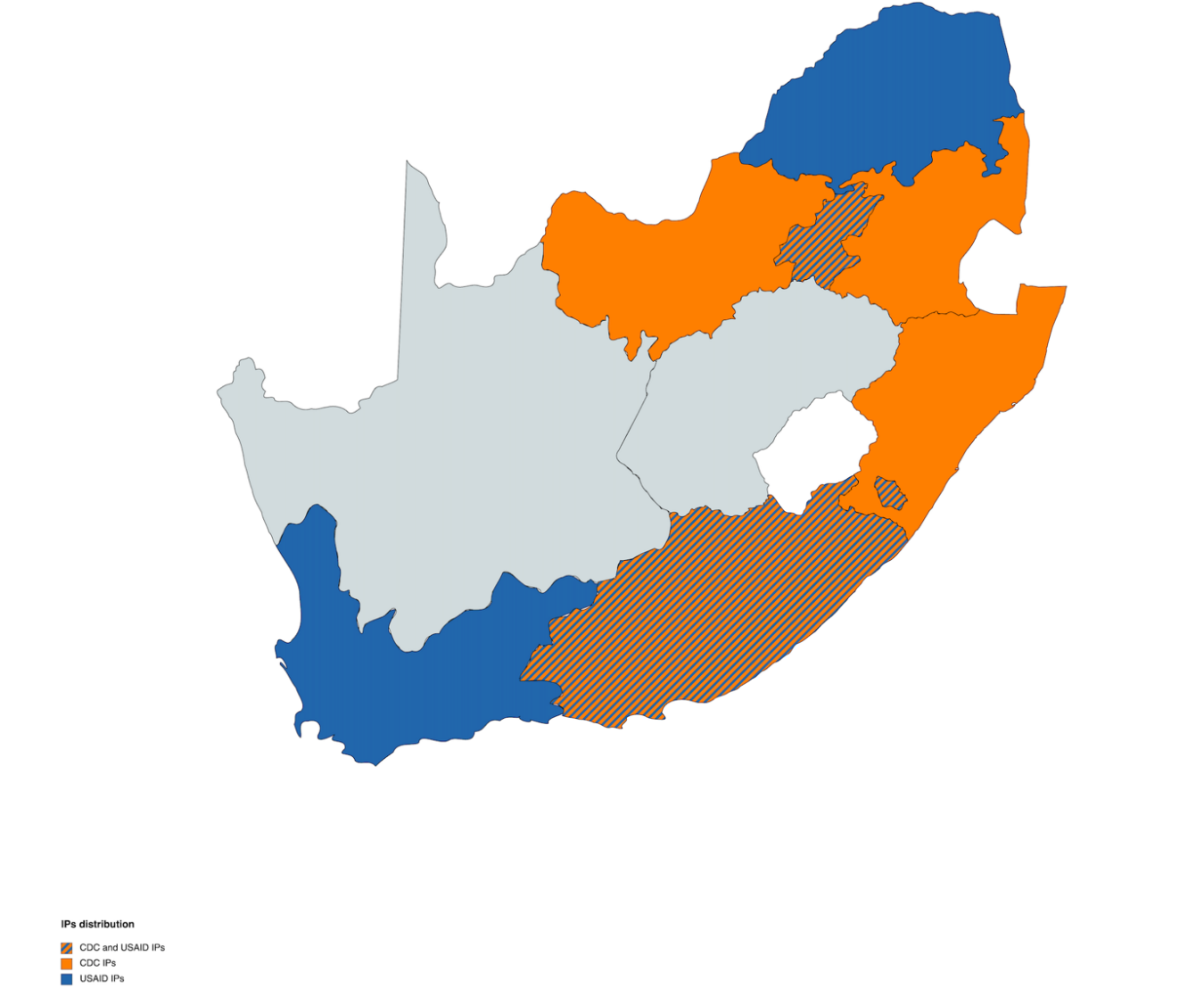

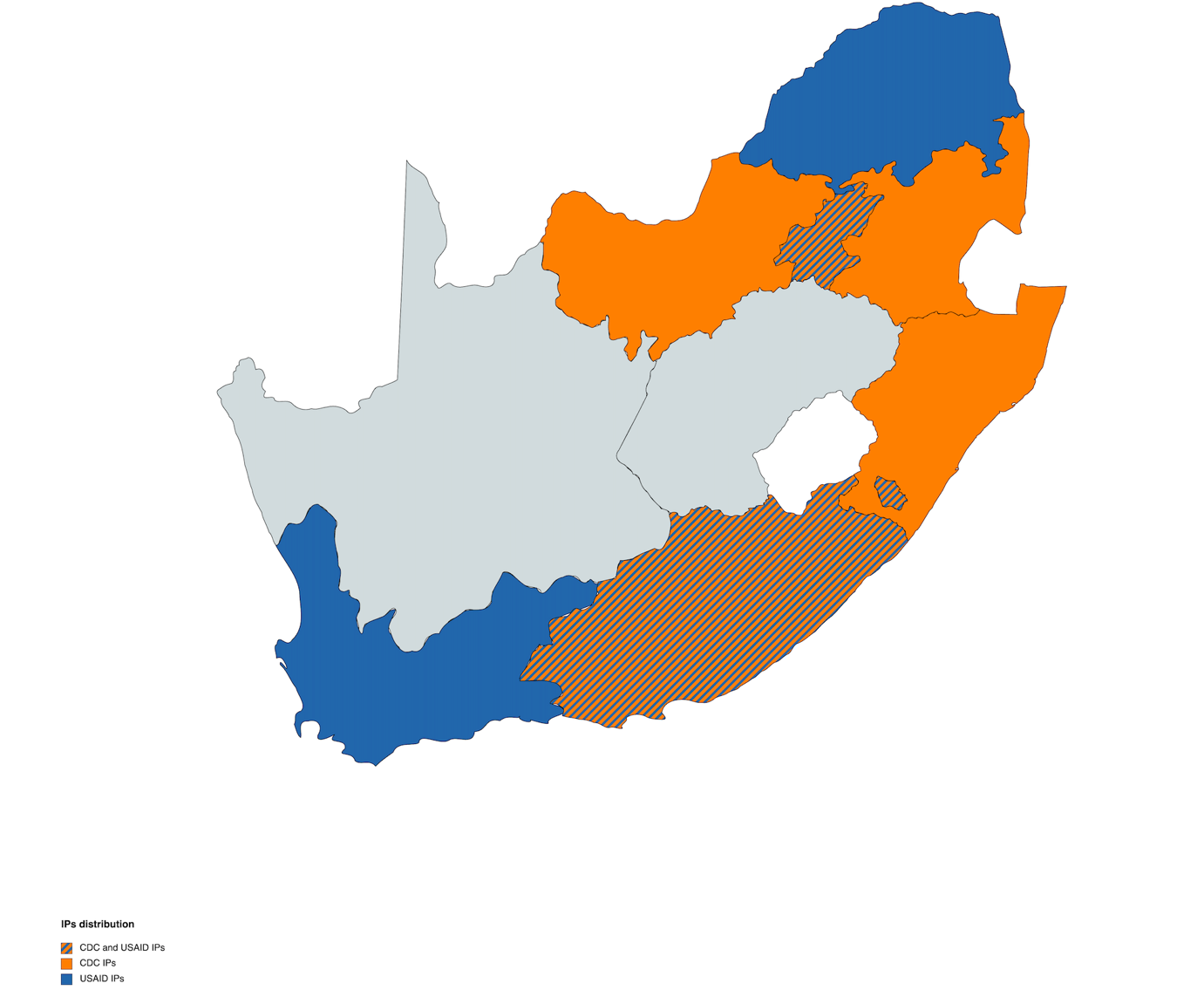

Supplement: Supplementary file 1 — Supplementary Material 1 [file 12889_2024_19679_MOESM1_ESM.docx]
